# Solid-State Construction of CuO_x_/Cu_1.5_Mn_1.5_O_4_ Nanocomposite with Abundant Surface CuO_x_ Species and Oxygen Vacancies to Promote CO Oxidation Activity

**DOI:** 10.3390/ijms23126856

**Published:** 2022-06-20

**Authors:** Baolin Liu, Hao Wu, Shihao Li, Mengjiao Xu, Yali Cao, Yizhao Li

**Affiliations:** 1 Yangtze Delta Region Institute (Huzhou), University of Electronic Science and Technology of China, Huzhou 313001, China; liubaolin6977@163.com; 2 State Key Laboratory of Chemistry and Utilization of Carbon Based Energy Resources, College of Chemistry, Xinjiang University, Urumqi 830017, China; xmj_1117@163.com; 3 College of Chemical Engineering, Xinjiang University, Urumqi 830046, China; wuhaode123@eyou.com (H.W.); lsh1531@126.com (S.L.)

**Keywords:** solid-state synthesis, CuO_x_/Cu_1.5_Mn_1.5_O_4_ nanocomposites, surface CuO_x_ species, oxygen vacancies, CO oxidation

## Abstract

Carbon monoxide (CO) oxidation performance heavily depends on the surface-active species and the oxygen vacancies of nanocomposites. Herein, the CuO_x_/Cu_1.5_Mn_1.5_O_4_ were fabricated via solid-state strategy. It is manifested that the construction of CuO_x_/Cu_1.5_Mn_1.5_O_4_ nanocomposite can produce abundant surface CuO_x_ species and a number of oxygen vacancies, resulting in substantially enhanced CO oxidation activity. The CO is completely converted to carbon dioxide (CO_2_) at 75 °C when CuO_x_/Cu_1.5_Mn_1.5_O_4_ nanocomposites were involved, which is higher than individual CuO_x_, MnO_x,_ and Cu_1.5_Mn_1.5_O_4_. Density function theory (DFT) calculations suggest that CO and O_2_ are adsorbed on CuO_x_/Cu_1.5_Mn_1.5_O_4_ surface with relatively optimal adsorption energy, which is more beneficial for CO oxidation activity. This work presents an effective way to prepare heterogeneous metal oxides with promising application in catalysis.

## 1. Introduction

Transition metal oxide catalysts for eliminating carbon monoxide (CO) at lower temperatures have attracted enormous attention in the past decades for their inexpensive cost and wide applications in catalytic applications and environmental protection [1,2,3]. Many techniques, such as morphology control [4,5,6,7], engineering defects [8,9,10], and construction of composite oxides [11,12,13] have been developed to improve the CO oxidation performance of transition metal oxide catalysts. Particularly, heterogeneous metal oxides that have exhibited excellent performances in CO oxidation fields [14,15,16] are the most widely studied because of their interactions of components [17]. Previous works have gradually demonstrated that heterogeneous metal oxides with synergistic interactions between two components are crucial for promoting catalytic performances [12,18,19]. The catalytic activity of nanocomposites depends significantly on surface active species and oxygen vacancies [20]. Therefore, the manipulation of the surface-active species and the oxygen vacancies of nanocomposites by simple strategy to optimize their catalytic performance is of great importance to meet the application in practice.

In the past few decades, many transition metal oxide catalysts have been developed, mainly including CeO_2_ [13,21,22], MnO_2_ [23,24,25], Co_3_O_4_ [26,27,28], CuO [29,30,31], Fe_2_O_3_ [32,33,34] et al., Co_3_O_4_-CeO_2−x_ [16,35], Cu-Mn [36], Ce-Cu [37,38], and Ce-Mn [39,40] composite oxides. Different active metals and carriers will result in different interactions between metals and carriers and different exposed active sites, thus making them have different reactivity for CO catalytic oxidation. Yu [16] synthesized the catalyst of Co_3_O_4_-CeO_2_ nanocomposite, which showed good catalytic activity due to its special hollow multishell structure and the interaction between the two components. Chen [41] constructed CuO_x_-CeO_2_ nanorods and explained the relationship between reduction treatment and catalytic activity, indicating that reduction treatment accelerates the generation of active sites. In our previous works, we fabricated a CuO_x_-CeO_2_ catalyst via the solid-state method [22], and investigated the influence of heating rate on catalytic performance. It was demonstrated that the heating rate can regulate the surface dispersion of CuO_x_ on CeO_2_ surface, resulting in enhanced catalytic performance. Copper-manganese mixed oxide catalyst is a typical transition metal-based catalyst in the CO oxidation reaction, which is known for its high activity at high temperature and low cost [36,42]. However, current commercial copper–manganese catalysts exhibit relatively low catalytic activity at low temperatures for CO oxidation. Furthermore, specific deactivation frequently occurs during the catalytic process [43,44]. The catalytic performance of heterogeneous catalysts is closely associated with their synergistic interactions and oxygen vacancies. In addition, many controllable synthetic strategies, such as direct calcination [30] and hydrothermal/solvothermal synthesis [45,46], have been developed for the fabrication of heterogeneous catalysts with the active two-phase interface, controllable size, shape, and composition in view of the purpose of improving catalytic performance. These synthetic routes are usually low-producing, time-consuming, and high-energy-consuming [47,48]. Solid-state synthesis integrated the advantages of low cost, eco-friendly and large-scale and have aroused wide concern in recent years [49,50,51,52,53].

Herein, a solid-state synthesis was developed to fabricate the CuO_x_/Cu_1.5_Mn_1.5_O_4_, which was implemented by the straightforward grinding of copper salt, manganese salt, and potassium hydroxide at ambient conditions. The metal oxide catalysts fabricated by solid-state synthesis are considered a simple and economical approach because they are without complicated procedures and organic solvents. The as-prepared CuO_x_/Cu_1.5_Mn_1.5_O_4_ exhibit significant advantages compared to other methods. The catalytic performance was obviously promoted, which can be attributed to the surface CuO_x_ species and the number of oxygen vacancies. More importantly, this work presents us with an effective way to prepare heterogeneous metal oxides with outstanding catalytic performance.

## 2. Results and Discussion

**Scheme 1:**
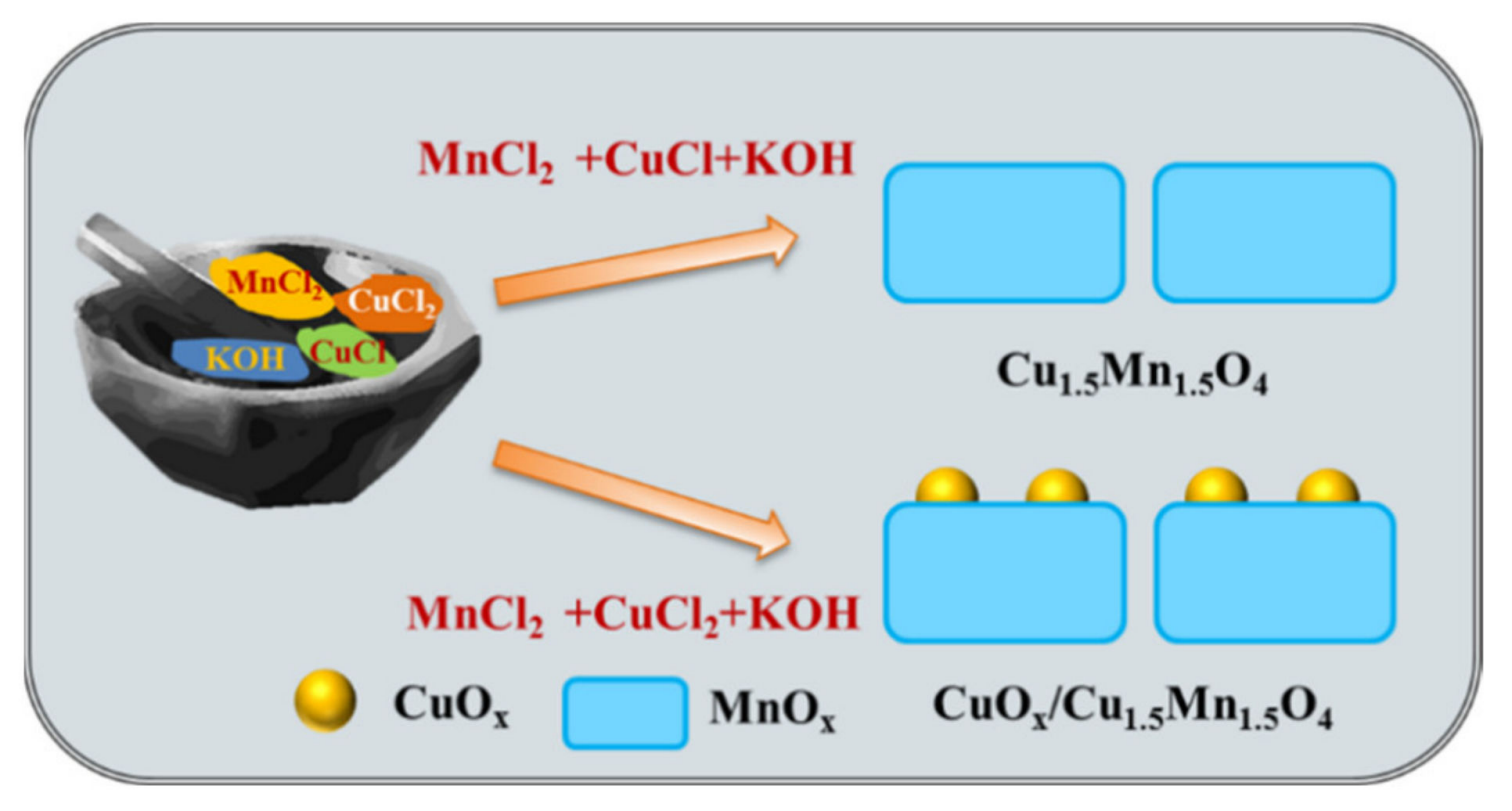
Schematic illustration of the construction of CuO_x_/Cu_1.5_Mn_1.5_O_4_ nanocomposite with different surface CuO_x_ species.

**Figure 1:**
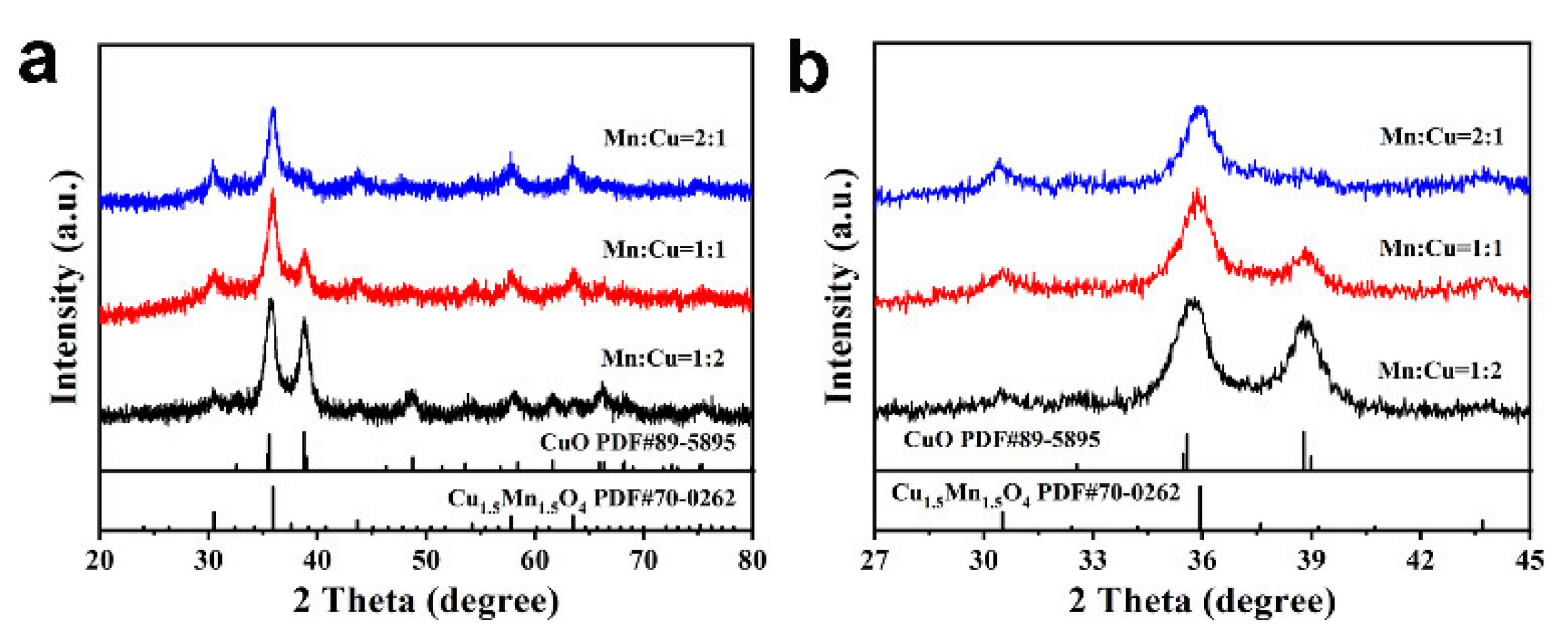
(**a**) The powder XRD patterns of CuO_x_/Cu_1.5_Mn_1.5_O_4_; (**b**) partially enlarged profiles.

The preparation process of CuO_x_/Cu_1.5_Mn_1.5_O_4_ is schematically illustrated in Scheme 1. The CuO_x_/Cu_1.5_Mn_1.5_O_4_ can be efficiently synthesized by the solvent-free strategy. The corresponding X-ray powder diffraction (XRD) patterns of CuO_x_/Cu_1.5_Mn_1.5_O_4_ are exhibited in Figure 1. The peaks detected at 2θ = 18.55, 30.51, 35.94, 37.60, 43.69, 54.23, 57.81, and 63.51 degrees were indexed to Cu_1.5_Mn_1.5_O_4_ spinel solid solution (JCPDS No:70-0262). The diffraction peak at 38.92 degrees attributes to the isolated CuO phases. These results demonstrate the successful synthesis of CuO_x_/Cu_1.5_Mn_1.5_O_4_ nanocomposites by solid-state strategy. As shown in Figure 1b, compared with individual CuO species, the major diffraction peaks of the products with different Cu/Mn mole ratios were slightly shifted to a higher degree, which can be ascribed to the change of the lattice parameters. In addition, XRD patterns show the characteristic peaks of CuO and MnO_x_, as shown in Figure S1. The ratios of Cu^2+^/Cu^+^ in Cu_2_O/CuO can be controlled by adjusting the types of copper salts. The energy-dispersive X-ray spectrum (EDS) mapping analyses was implemented to identify the elementary composition of the CuO_x_/Cu_1.5_Mn_1.5_O_4_. The Cu/Mn molar ratio in the as-prepared samples is close to 1.0 (Figure S2), which has no significant difference compared to the theoretical value during synthesis.

**Figure 2:**
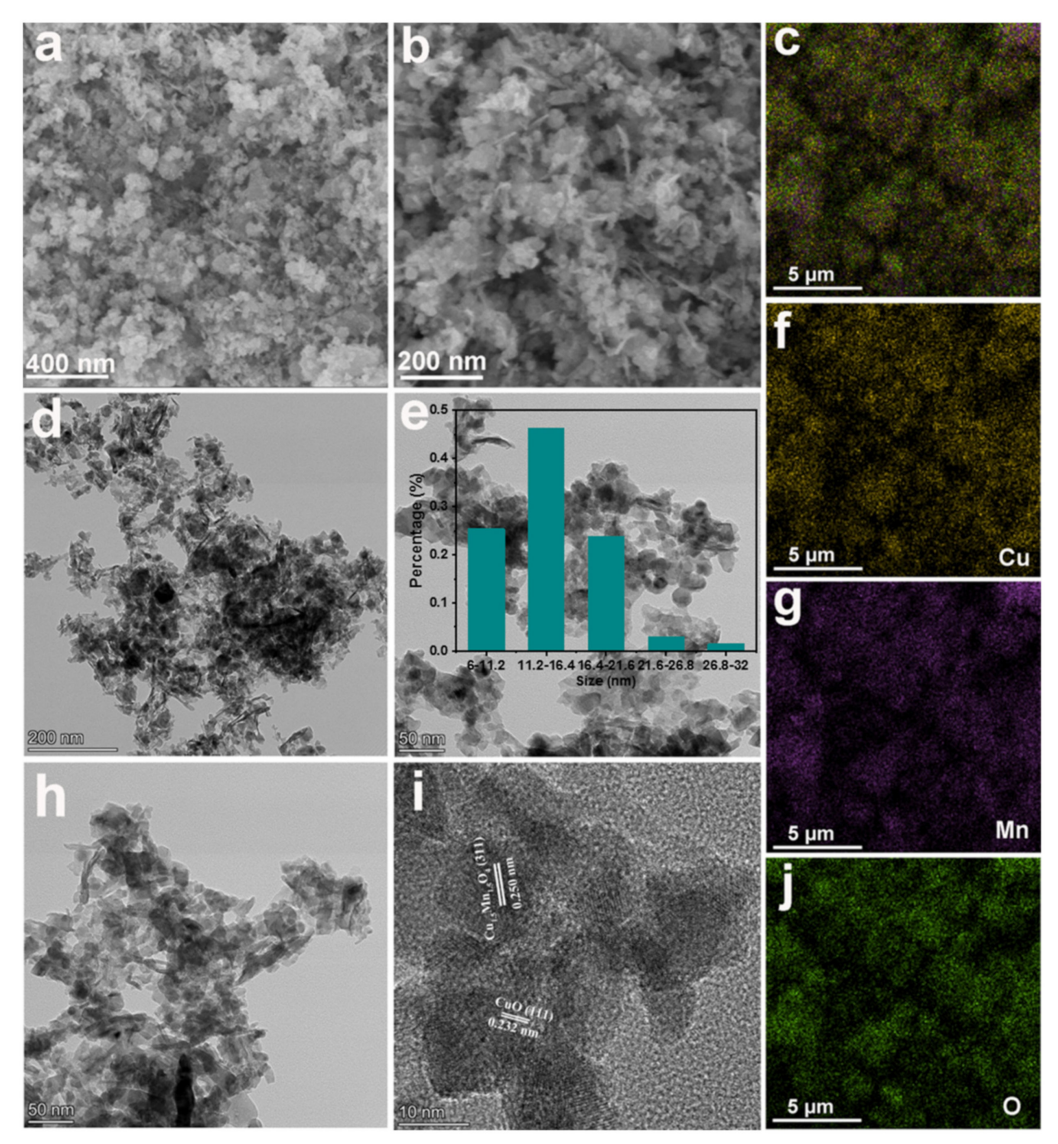
(**a**,**b**) the SEM images; (**d**,**e**,**h**) TEM images; (**i**) HRTEM image; and (**c**,**f**,**g**,**j**) the corresponding element mapping patterns of CuO_x_/Cu_1.5_Mn_1.5_O_4_.

The scanning electron microscope (SEM) and transmission electron microscopes (TEM) images as shown in Figure 2 further prove that the nanoparticles with 11–16 nm (Figure 2e) existed in CuO_x_/Cu_1.5_Mn_1.5_O_4_. Additionally, the interplanar spacing of 0.250 nm and 0.232 nm, revealed by the high-resolution transmission electron microscopes (HRTEM) image (Figure 2i), correspond to (311) and (111) planes of Cu_1.5_Mn_1.5_O_4_ and CuO, respectively. The element mapping results are exhibited in Figure 2, confirming that the Cu and Mn elements are uniformly dispersed on the surface of CuO_x_/Cu_1.5_Mn_1.5_O_4_. These results further confirm the successful synthesis of CuO_x_/Cu_1.5_Mn_1.5_O_4_ by solid-state strategy. Furthermore, the morphologies of CuO_x_/Cu_1.5_Mn_1.5_O_4_ prepared by adjusting the molar ratios of Cu/Mn were also acquired (Figure S3), displaying agglomerated nanoparticles. The morphologies of CuO, Cu_2_O-CuO, and MnO_x_ are shown in Figures S4–S6, which also exhibit ir-regular nanoparticles.

**Figure 3:**
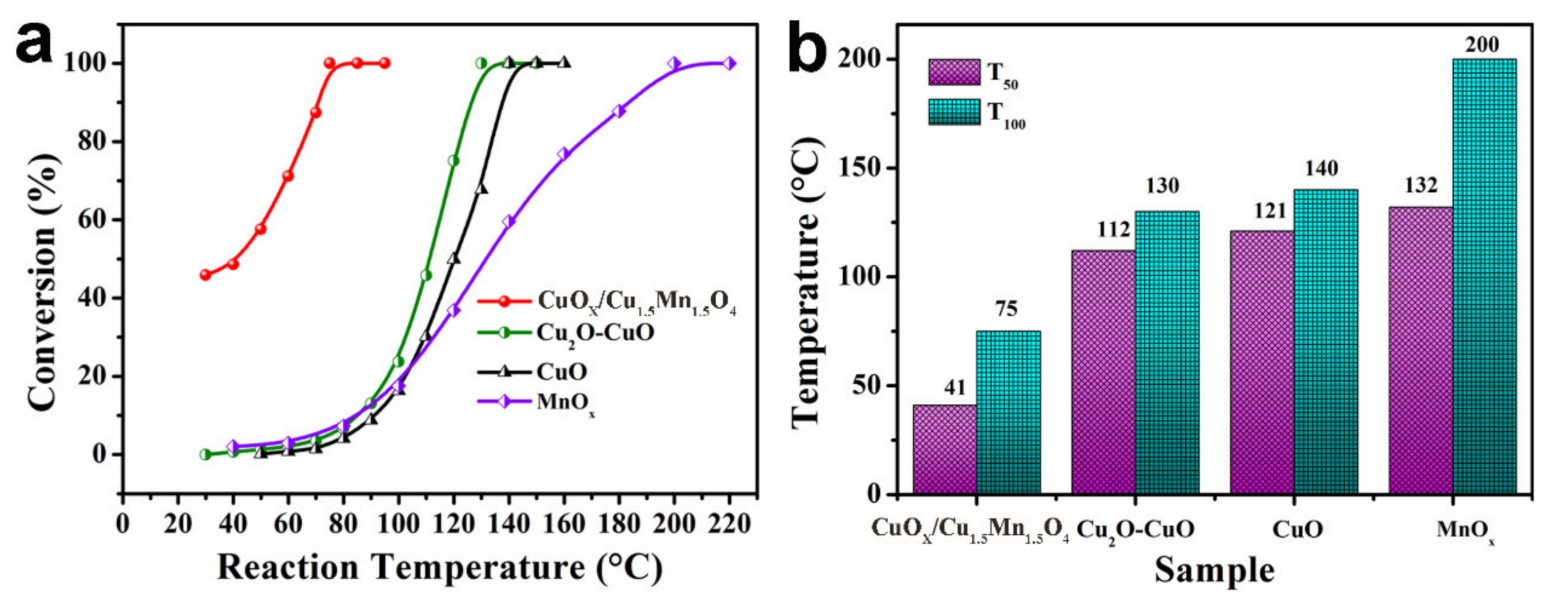
(**a**,**b**) The CO conversion performances on various catalysts.

The CO catalytic performance of the as-obtained CuO_x_/Cu_1.5_Mn_1.5_O_4_ were firstly evaluated. As shown in Figure S7a,b, the best CO catalytic activity is CuO_x_/Cu_1.5_Mn_1.5_O_4_ with Cu/Mn molar ratio of 1:1 calcined at 400 °C. The CuO_x_/Cu_1.5_Mn_1.5_O_4_ can completely convert CO to CO_2_ at 75 °C, especially at low temperatures. T_50_ (the temperature of 50% of CO conversion) is only 41 °C. The CO_2_ yield in the CO oxidation has been shown in Figure S8a, which presents nearly 100% yield. Moreover, they have been compared with previous works (Table S1), which also presents a better catalytic property. Other samples exhibit a relatively lower catalytic activity performance with higher T_100_ (the temperature of 100% of CO conversion) and T_50_ (the temperature of 50% of CO conversion) in Figure 3. The 100% CO conversion was accomplished for individual CuO, Cu_2_O-CuO, and MnO_x_ samples at 140 °C, 130 °C, and 200 °C, respectively. The individual CuO_x_ and MnO_x_ particles show poorer performance than CuO_x_/Cu_1.5_Mn_1.5_O_4_, implying that the synergistic effect between CuO_x_ and MnO_x_ may promote its catalytic activity that is not presented in the individual components. As shown in Figure S8b, the sample of physical mixing of CuO_x_ + MnO_x_ was also prepared, which exhibits the poor catalytic performance for CO oxidation. The stability of the CuO_x_/Cu_1.5_Mn_1.5_O_4_ was also tested at 60 °C. The negligible decline of activity can be observed during 30 h testing from Figure S9, which implies the excellent stability of CuO_x_/Cu_1.5_Mn_1.5_O_4_ for CO oxidation reaction.

**Figure 4:**
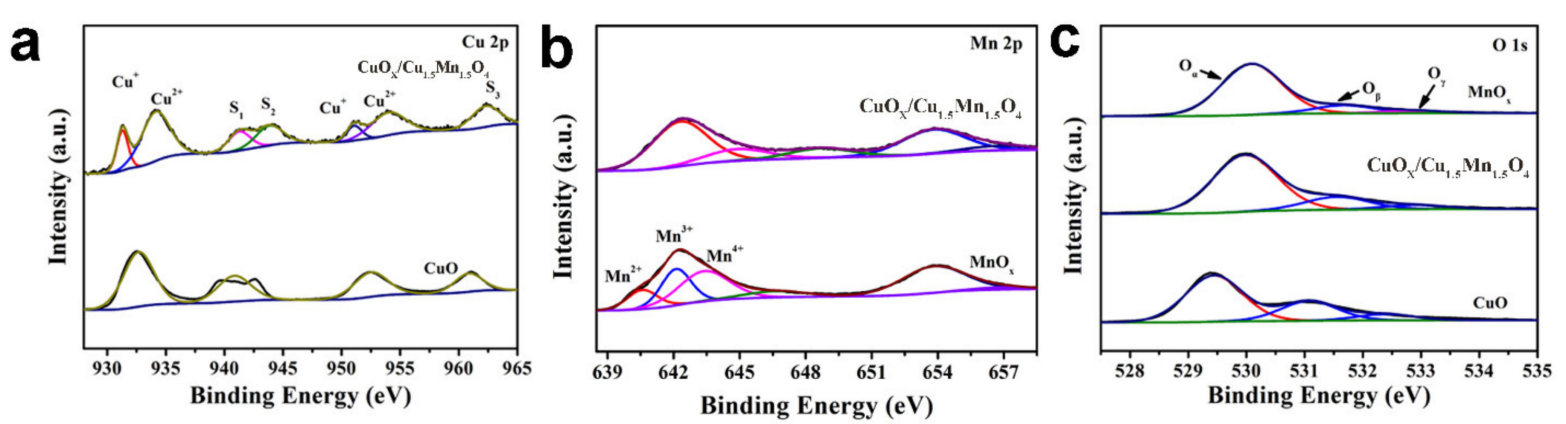
(**a**) Cu 2p; (**b**) Mn 2p; (**c**) O 1s spectra of MnO_x_, CuO_x_/Cu_1.5_Mn_1.5_O_4_, and CuO catalysts, respectively.

The X-ray photoelectron spectra (XPS) were used to investigate the chemical states of samples. The XPS spectrum in Figure 4 and Figure S10 indicate the coexistence of the Cu, Mn, and O elements. Two peaks at about 931.1 and 950.9 eV, respectively, shown in Figure 4a refer to the Cu^+^ or Cu° due to the fact that their binding energies are basically the same [31,54]. Cu° is unstable at room temperature and easily oxidized to copper oxide. The CuO_x_/Cu_1.5_Mn_1.5_O_4_ nanocomposites were acquired after being calcined at high temperature in air. Therefore, the peak is assigned to Cu^+^ because of the successful synthesis of CuO_x_/Cu_1.5_Mn_1.5_O_4_ nanocomposites. The two main peaks have small shoulder peaks that appeared at 933.3 and 953.4 eV, relating to the Cu^2+^ [55,56,57,58]. The XPS analysis results imply that Cu^+^ and Cu^2+^ coexist on the surfaces of the CuO_x_/Cu_1.5_Mn_1.5_O_4_. As shown in Figure 4b, the asymmetrical Mn 2p spectra of individual MnO_x_ catalysts could be fitted into four components based on their binding energies. The binding energies of 640.2 eV, 641.2 eV, 642.5 eV, and 646.0 eV correspond to Mn^2+^, Mn^3+^, Mn^4+^ species, and the satellite peak, respectively [59]. The O 1s XPS spectrum of samples can be divided into three single peaks (Figure 4c), corresponding to surface lattice oxygen (O_α_), surface adsorbed oxygen (O_β_), and adsorbed molecular water species (O_γ_), respectively [14,60,61,62]. The CuO_x_/Cu_1.5_Mn_1.5_O_4_ demonstrates the highest surface adsorbed oxygen, which is beneficial to the adsorption of O_2_ molecules, and thus help to improve catalytic performance.

**Figure 5:**
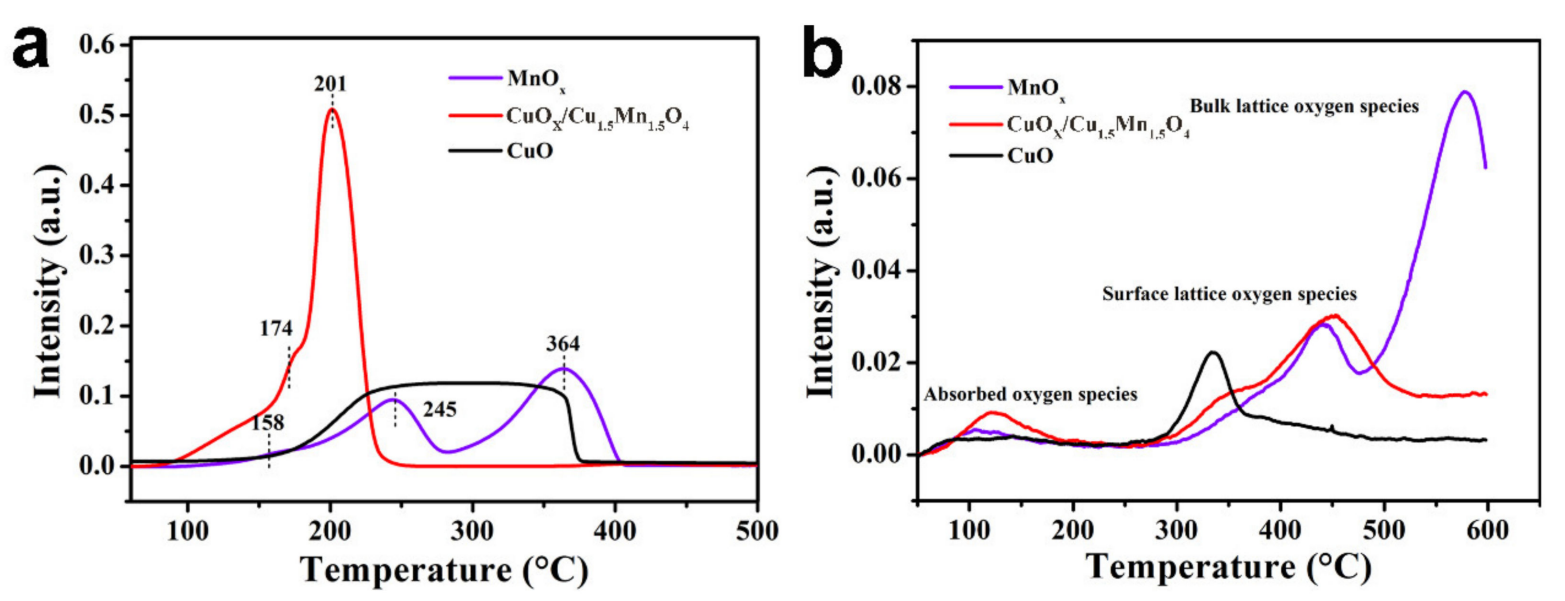
(**a**) H_2_-TPR; (**b**) O_2_-TPD profiles of MnO_x_, CuO_x_/Cu_1.5_Mn_1.5_O_4_, and CuO catalysts, respectively.

As shown in Figure 5a, the reduction property of prepared samples was investigated by hydrogen temperature-programmed reduction (H_2_-TPR). A peak at 200 °C–400 °C was presented for an individual CuO sample, attributing to the gradual reduction of copper oxide [30]. In addition, two H_2_ reduction peaks at 245 °C and 364 °C occurred for an MnO_x_ sample, which correspond to the gradual reduction of MnO_2_ → Mn_3_O_4_ → MnO [23,39,59]. For CuO_x_/Cu_1.5_Mn_1.5_O_4_ nanocomposite, the first peak at below 158 °C refers to the reduction of fine CuO to Cu or MnO_2_ to Mn_3_O_4_. Other peaks at 174 °C and 201 °C correspond to the gradual reduction of Cu_1.5_Mn_1.5_O_4_ oxides [63]. The lower reduction temperature for CuO_x_/Cu_1.5_Mn_1.5_O_4_ compared with individual CuO_x_ and MnO_x_ indicates the strong synergistic effect between CuO_x_ and MnO_x_. The strong interactions in CuO_x_/Cu_1.5_Mn_1.5_O_4_ are often related to the abundant oxygen vacancies, which can promote catalytic performance. The oxygen storage capacity (OSC) of catalysts was assessed by oxygen temperature-programmed desorption (O_2_-TPD). In Figure 5b, the temperature below 200 °C is due to the desorption of surface oxygen species (O_β_) [64]. The second peak, appearing at 250–550 °C, corresponds to the overflow of surface lattice oxygen (O_α_). The high-temperature zone at above 550 °C is related to the bulk lattice oxygen species [63]. The CuO_x_/Cu_1.5_Mn_1.5_O_4_ shows the highest amount of adsorbed oxygen species compared with other samples, confirming the higher oxygen capacity that is conducive to the promotion of catalytic performance.

**Figure 6:**
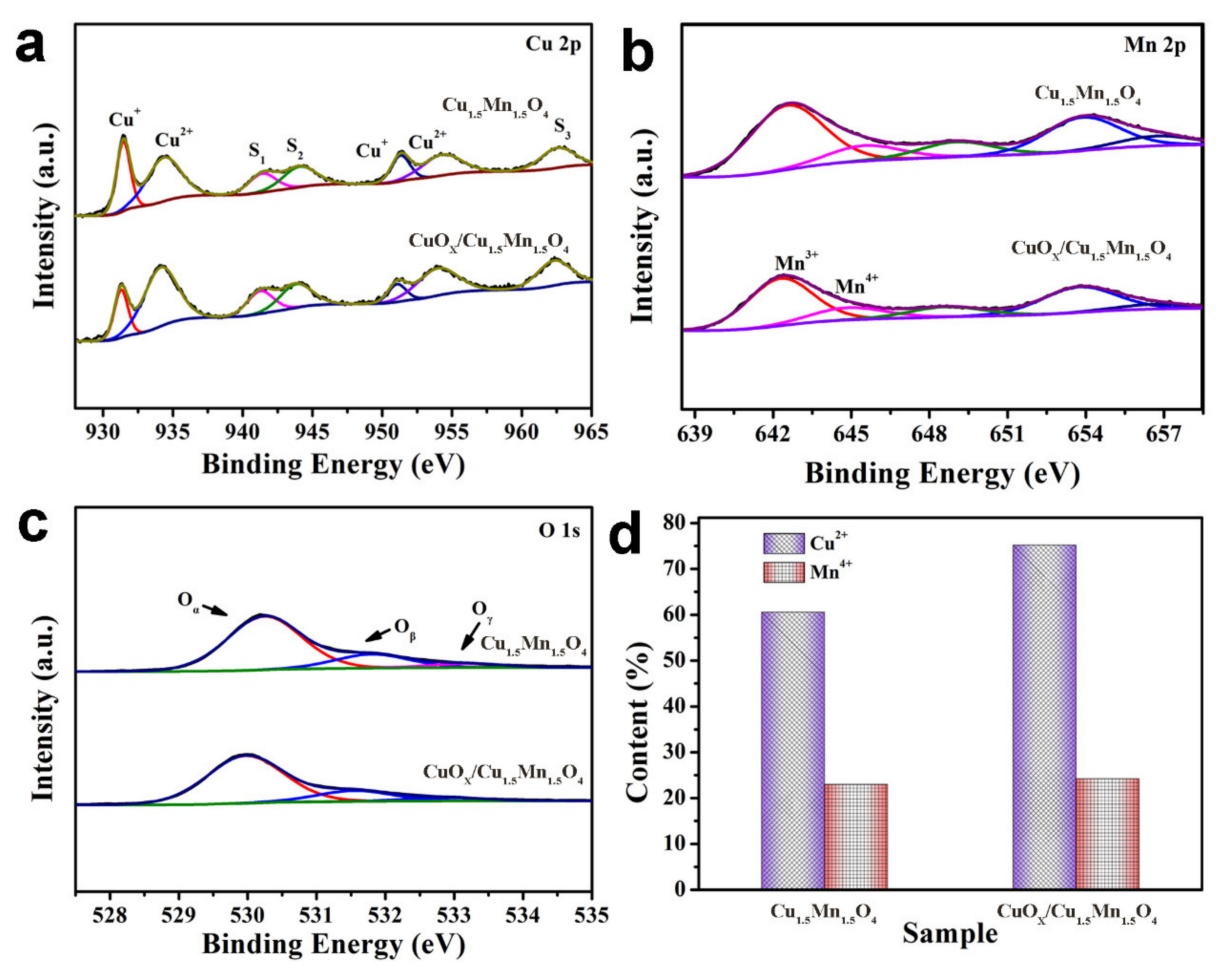
(**a**) Cu 2p; (**b**) Mn 2p; (**c**) O 1s spectra; (**d**) the contents of Cu^2+^ and Mn^4+^ in Cu_1.5_Mn_1.5_O_4_, and CuO_x_/Cu_1.5_Mn_1.5_O_4_, respectively.

To elucidate the effects of surface CuO_x_ species and the oxygen vacancies in CuO_x_/Cu_1.5_Mn_1.5_O_4_ in detail, the Cu_1.5_Mn_1.5_O_4_ was fabricated by changing the types of copper salt in the synthesis process to investigate the structure-activity relationships. The Cu^2+^ and Mn^4+^ proportion was evaluated by XPS (Figure 6d). It indicates that the ratio of Cu^2+^ and Mn^4+^ in CuO_x_/Cu_1.5_Mn_1.5_O_4_ is higher than Cu_1.5_Mn_1.5_O_4_ sample. The higher contents of Cu^2+^ and Mn^4+^ are beneficial to the formation of Cu^2+^-O^2−^Mn^4+^ entities at the two-phase interface [36]. As reported in the studies [63,65], the presence of abundant Mn^4+^ proportion can create many adsorbed oxygen species [63]. In addition, the ratios of Cu^+^/Cu^2+^ in different samples show a change by altering the types of copper salts in the synthetic process (Table S2). The CuO_x_/Cu_1.5_Mn_1.5_O_4_ exhibits a higher Cu^2+^ ratio than Cu_1.5_Mn_1.5_O_4_, which corresponds to XRD results that the CuO_x_/Cu_1.5_Mn_1.5_O_4_ shows the higher intensity of the CuO diffraction peaks. The XPS results indicate that the Cu^2+^ and Mn^4+^ proportion can be engineered by changing the component of Cu-based oxides in the synthetic process.

**Figure 7:**
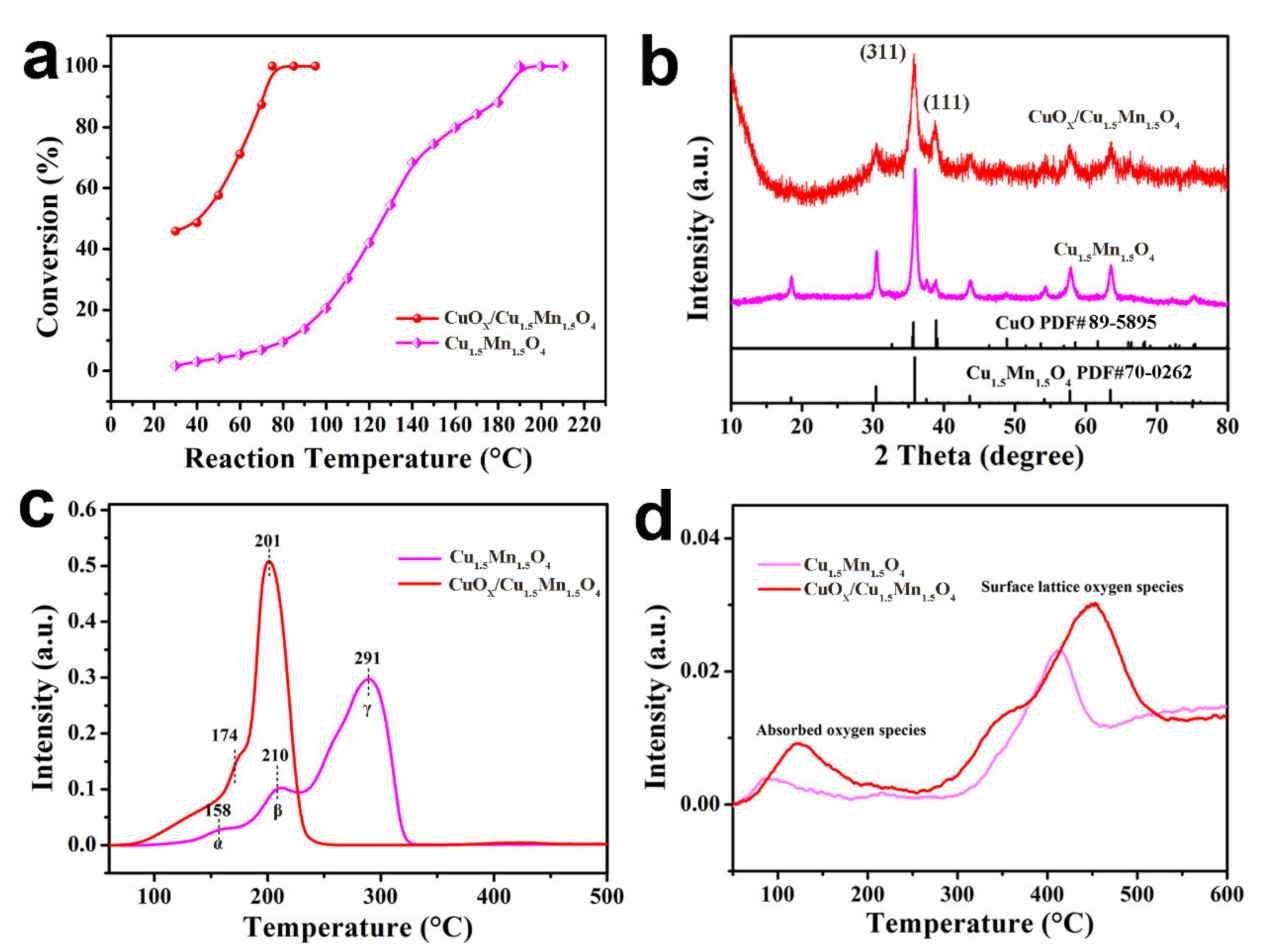
(**a**) The CO conversion performances; (**b**) the powder XRD patterns; (**c**) H_2_-TPR; (**d**) O_2_-TPD profiles of Cu_1.5_Mn_1.5_O_4_ and CuO_x_/Cu_1.5_Mn_1.5_O_4_, respectively.

As shown in Figure 7a, there are obvious differences in catalytic performance after changing the copper salts. The Cu_1.5_Mn_1.5_O_4_ exhibits poor catalytic activity compared with CuO_x_/Cu_1.5_Mn_1.5_O_4_ at the same condition. Herein, the performance of catalysts can be meaningfully boosted by altering the surface CuO_x_ species and the oxygen vacancies in CuO_x_/Cu_1.5_Mn_1.5_O_4_. As shown in Figure S11a,b, the Cu_1.5_Mn_1.5_O_4_ exhibited ir-regular nanoparticles morphology. The Cu and Mn elements are homogeneously dispersed on the surface of Cu_1.5_Mn_1.5_O_4_, and their molar ratio is close to 1.0 (Figure S2), which has no significant difference with CuO_x_/Cu_1.5_Mn_1.5_O_4_. The XRD diffraction patterns indicate the formation of Cu_1.5_Mn_1.5_O_4_ containing some CuO_x_ (Figure 7b), while the catalytic performance showed obvious differences. The CuO_x_/Cu_1.5_Mn_1.5_O_4_ exhibits the higher intensity of the CuO diffraction peaks than Cu_1.5_Mn_1.5_O_4_. In our previous work [31], the individual Cu_2_O/CuO nanocomposites and CuO can be fabricated by tuning the types of copper salt in the synthesis. The Cu_1.5_Mn_1.5_O_4_ with different surface CuO_x_ types were fabricated by altering the types of copper salts (+1, +2 valence state) in the synthetic process. Therefore, the different surface types in Cu_1.5_Mn_1.5_O_4_ depend on the types of copper salts (+1, +2 valence state) in the synthetic process used. The higher content of CuO in CuO_x_/Cu_1.5_Mn_1.5_O_4_ can significantly enhance redox reaction between Cu and Mn species, promoting charge transfer in nanocomposites, and thus achieving a stronger interaction. From Figure 7c, the CuO_x_/Cu_1.5_Mn_1.5_O_4_ shows a lower reduction temperature than Cu_1.5_Mn_1.5_O_4_, implying the better reducibility. The peak areas for different samples were estimated from the H_2_-TPR results. In Table S3, the higher peak areas of first peak α is presented for CuO_x_/Cu_1.5_Mn_1.5_O_4_, indicating the ratio of Cu^2+^ and Mn^4+^ in CuO_x_/Cu_1.5_Mn_1.5_O_4_, which is consistent with XPS results. Therefore, we can confirm that CO oxidation activity is heavily dependent on the surface CuO_x_ species in CuO_x_/Cu_1.5_Mn_1.5_O_4_. In addition, as shown in Figure 7d, the amounts of oxygen desorption (O_β_) over the obtained CuO_x_/Cu_1.5_Mn_1.5_O_4_ are greatly changed by adjusting the surface CuO_x_ species. The CuO_x_/Cu_1.5_Mn_1.5_O_4_ shows the highest amount of adsorbed oxygen species compared with Cu_1.5_Mn_1.5_O_4_, confirming the higher oxygen capacity that is conducive to the promotion of catalytic performance. The H_2_-TPR analysis results indicate that the interaction of nanocomposites could be manipulated by changing the surface compositions of CuO_x_/Cu_1.5_Mn_1.5_O_4_. The O_2_-TPD results further identify the presence of abundant surface-adsorbed oxygen on the surface of CuO_x_/Cu_1.5_Mn_1.5_O_4_. Therefore, the construction of CuO_x_/Cu_1.5_Mn_1.5_O_4_ with abundant surface CuO_x_ species not only strengthens the interactions in CuO_x_/Cu_1.5_Mn_1.5_O_4_, but also facilitates the absorption and activation of surface oxygen species.

**Figure 8:**
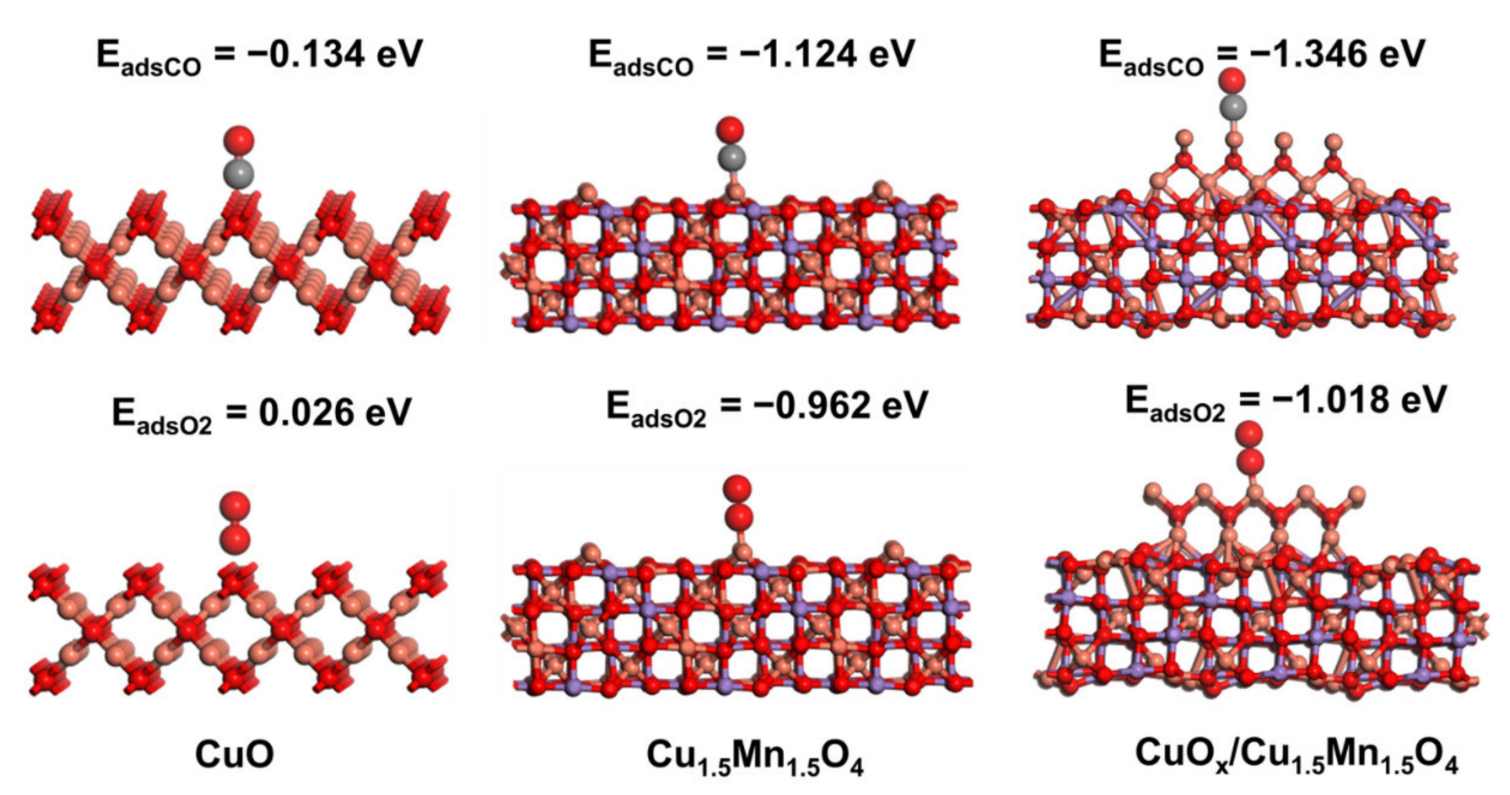
The side views of CO and O_2_ adsorption on the surfaces of CuO, Cu_1.5_Mn_1.5_O_4_, and CuO_x_/Cu_1.5_Mn_1.5_O_4_, respectively.

Density functional theory (DFT) calculations were implemented to understand the intrinsic reason about the mechanism of CO and O_2_ adsorption and the subsequent oxidation process for CuO_x_/Cu_1.5_Mn_1.5_O_4_. The adsorption configurations of CO and O_2_ molecules on the CuO, Cu_1.5_Mn_1.5_O_4_, and CuO_x_/Cu_1.5_Mn_1.5_O_4_ are shown in Figure 8. It is found that CO is adsorbed on the surface of CuO and Cu_1.5_Mn_1.5_O_4_ with the adsorption energy of −0.316 eV and −0.164 eV, respectively. The adsorption energy of CO molecules adsorbed at the CuO_x_/Cu_1.5_Mn_1.5_O_4_ surface is −1.346 eV, which are lower than that on CuO and Cu_1.5_Mn_1.5_O_4_. In addition, the adsorption energy of O_2_ molecules on CuO_x_/Cu_1.5_Mn_1.5_O_4_ surface is −1.018 eV, which is much lower than the 0.026 eV and −0.962 eV for CuO and Cu_1.5_Mn_1.5_O_4_. The lower adsorption energy indicates that gas molecules are easier to adsorb on the surface of CuO_x_/Cu_1.5_Mn_1.5_O_4_. In summary, DFT calculation showed that construction of CuO_x_/Cu_1.5_Mn_1.5_O_4_ nanocomposite with abundant surface CuO_x_ species and oxygen vacancies significantly improved the adsorption capacity of CO and O_2_ molecules, and thus is more beneficial for CO oxidation activity.

**Figure 9:**
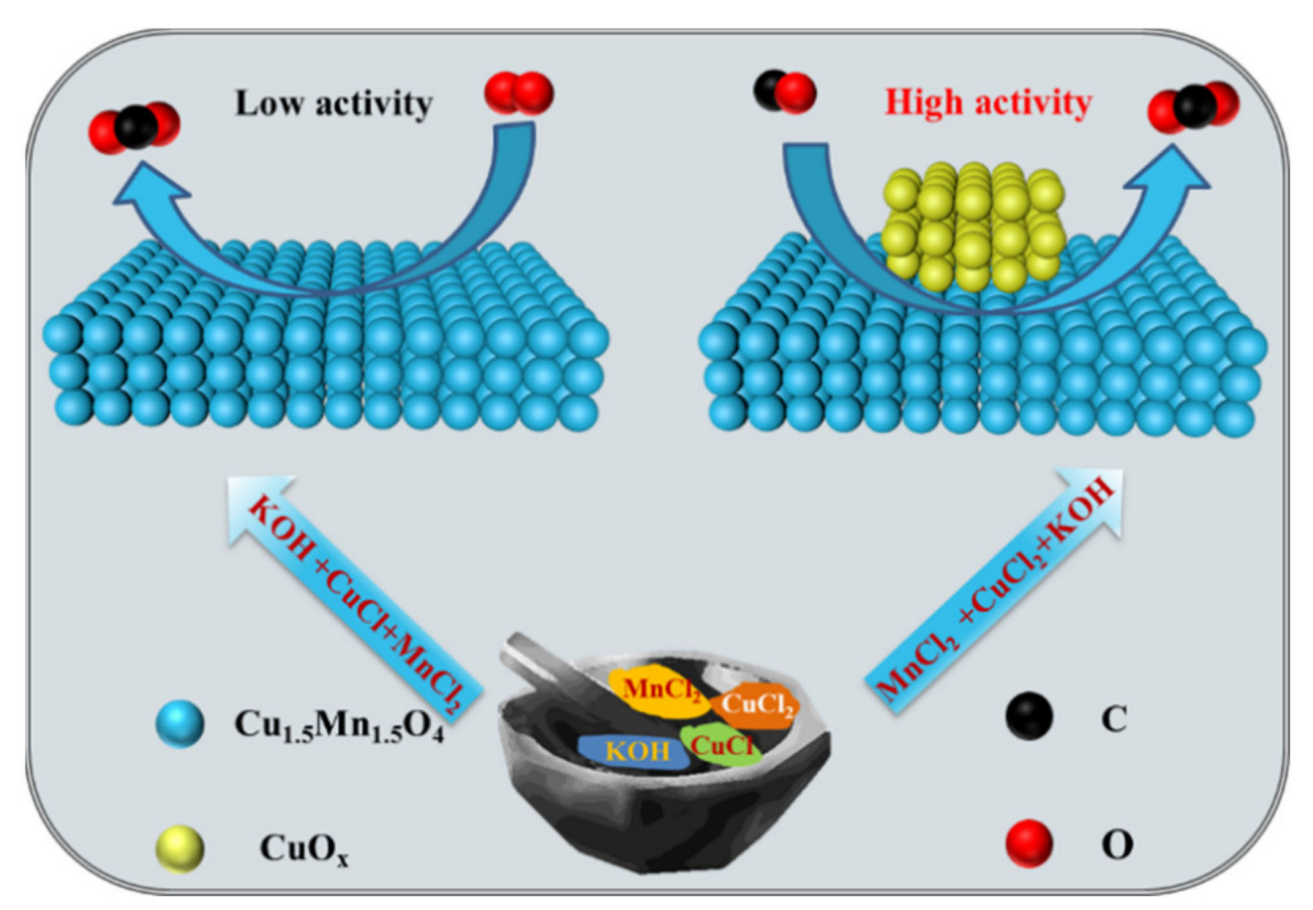
Reaction mechanisms of CuO_x_/Cu_1.5_Mn_1.5_O_4_ toward CO oxidation.

The effects of surface CuO_x_ species and the oxygen vacancies in composite oxide can be clarified based on the above results. In Figure 9, the catalytic property of the CuO_x_/Cu_1.5_Mn_1.5_O_4_ is improved compared to Cu_1.5_Mn_1.5_O_4_, which confirms the important role of surface CuO_x_ species and oxygen vacancies. After construction of CuO_x_/Cu_1.5_Mn_1.5_O_4_ nanocomposite with abundant surface CuO_x_ species and oxygen vacancies, the abundant Cu^2+^ and Mn^4+^ proportions in CuO_x_/Cu_1.5_Mn_1.5_O_4_ are higher than in Cu_1.5_Mn_1.5_O_4_, which facilitated the formation of more (Cu^2+^-O^2−^-Mn^4+^) entities at the two interfaces. In addition, the construction of CuO_x_/Cu_1.5_Mn_1.5_O_4_ nanocomposites is beneficial for enhancing the synergetic interaction between MnO_x_ species and CuO_x_ species, which promotes the massive production of surface adsorbed oxygen species [36]. In CO oxidation, surface CuO_x_ species and oxygen vacancies play significant roles in catalytic activity. The abundant surface CuO_x_ species and oxygen vacancies could preferentially adsorb CO and O_2_ molecules [22], and the adsorbed O_2_ reacts with CO to form CO_2_, which ultimately enhances catalytic activity.

## 3. Materials and Methods

### 3.1. Materials

Copper (II) chloride (CuCl_2_, AR), cuprous (I) chloride (CuCl, AR), manganese (II) chloride (MnCl_2_, AR), and potassium hydroxide (KOH, AR) were purchased from Tianjin Zhiyuan Chemical Reagents Co., Ltd. (Tianjin, China), which were used without further refinement.

### 3.2. The Preparation of CuO_x_/Cu_1.5_Mn_1.5_O_4_ Nanocomposite with Various Surface CuO_x_ Species and the Oxygen Vacancies

As shown in Scheme 1, in a typical procedure, 1.70 g of CuCl_2_ (10 mmol) and 1.25 g of MnCl_2_ (10 mmol) were mixed well in an agate mortar by grinding. Then 4.49 g of KOH (60 mmol) was added into the agate mortar. After continuous grinding for about 1 h, the resulting solid products were sufficiently washed with deionized water and anhydrous ethanol to clear the residual Cl or K species, and then dried at ambient temperature overnight. The final CuO_x_/Cu_1.5_Mn_1.5_O_4_ nanocomposites were acquired after calcining the mixtures in the air at 400 °C for 2 h (5 °C/min). In addition, the CuO_x_/Cu_1.5_Mn_1.5_O_4_ with different Cu/Mn mole ratios (Cu/Mn = 1:2 and 2:1) were calcined at 300 °C or 500 °C.

The Cu_1.5_Mn_1.5_O_4_ nanocomposite (containing some CuO) was also obtained, and only the CuCl_2_ was replaced by CuCl during the solvent-free synthesis route.

### 3.3. The Preparation of MnO_x_

As a comparison, the individual MnO_x_ particles were also fabricated by straightforward grinding MnCl_2_ with KOH under a similar process.

### 3.4. The Preparation of Cu_2_O/CuO and CuO

The Cu_2_O/CuO nanocomposite was fabricated according to our previous work [31]. The 0.99 g of CuCl (10 mmol) and 1.68 g of KOH (30 mmol) were ground in the agate mortar for 1 h. The other parameters are consistent with the CuO_x_/Cu_1.5_Mn_1.5_O_4_ nanocomposite above.

In addition, the CuO was also prepared by mixing CuCl_2_ and KOH in the agate mortar. The sample of physical mixing of CuO_x_ + MnO_x_ was also prepared by straightforward grinding CuO and MnO_x_, and then calcining the mixtures in the air at 400 °C for 2 h (5 °C/min).

### 3.5. The Characterization and Testing Processes of Catalyst

XRD, SEM, HRTEM, EDS, XPS, H_2_-TPR, and O_2_-TPD were implemented to investigate the morphology and structure of CuO_x_/Cu_1.5_Mn_1.5_O_4_ nanocomposites. Detailed characterization and testing processes are presented in the Supplementary Materials.

## 4. Conclusions

In summary, the Cu_1.5_Mn_1.5_O_4_ with different surface CuO_x_ types were fabricated by altering the types of copper salts (+1, +2 valence state) in the synthetic process. The higher content of CuO in CuO_x_/Cu_1.5_Mn_1.5_O_4_ can significantly enhance redox reaction between Cu and Mn species, promoting charge transfer in nanocomposites, thus achieving a stronger interaction. In addition, the higher ratio of Cu^2+^ and Mn^4+^ is beneficial to the formation of Cu^2+^-O^2−^-Mn^4+^ entities at the two-phase interface, which produced abundant surface CuO_x_ species and oxygen vacancies. DFT calculations suggest that CO and O_2_ molecules are adsorbed on the CuO_x_/Cu_1.5_Mn_1.5_O_4_ surface with relatively optimal adsorption energy, resulting in the highest CO oxidation activity. The as-synthesized CuO_x_/Cu_1.5_Mn_1.5_O_4_ delivers excellent CO catalytic performance compared with individual CuO_x_ and MnO_x_ particles. The CO is completely converted to CO_2_ at 75 °C when CuO_x_/Cu_1.5_Mn_1.5_O_4_ is involved. This work opens new avenues for the efficient and sustainable production of heterogeneous metal oxides with an outstanding catalytic performance.

## Data Availability

The study did not report any data, we choose to exclude this statement.

## Supplementary Materials

The following supporting information can be downloaded at: https://www.mdpi.com/article/10.3390/ijms23126856/s1. References [66,67,68] are cited in Supplementary Materials.
