# Supplementary material for: Solid-State Construction of CuOx/Cu1.5Mn1.5O4 Nanocomposite with Abundant Surface CuOx Species and Oxygen Vacancies to Promote CO Oxidation Activity
Source: Int J Mol Sci. 2022 Jun 20;23(12):6856. doi: 10.3390/ijms23126856 (PMC9224245; doi:10.3390/ijms23126856)
Supplement: Supplementary file 1 [file ijms-23-06856-s001.zip › ijms-1759012-supplementary.pdf]

Supporting information

Solid-State Construction of $CuO_x/Cu_{1.5}Mn_{1.5}O_4$ Nanocomposite with Abundant Surface CuO_x Species and Oxygen Vacancies to Promote CO Oxidation Activity

Baolin Liu[2,†], Hao Wu[3,†], Shihao Li[3], Mengjiao Xu[2], Yali Cao[2,*] , Yizhao Li[1,3,*]

[1] *Yangtze Delta Region Institute (Huzhou), University of Electronic Science and Technology of China, Huzhou 313001, China.*

[2] *State Key Laboratory of Chemistry and Utilization of Carbon Based Energy Resources; College of Chemistry, Xinjiang University, Urumqi, 830017, Xinjiang, PR China;*

[3] *College of Chemical Engineering, Xinjiang University, Urumqi 830046, China.*

[†] *These authors contributed equally to this work.*

**Corresponding author. Tel: +86-572-2370780; Fax: +86-572-2370780;*

E-mails: liyizhao0809@126.com (Y. Li); caoyali523@163.com (Y. Cao).

1. The characterization of catalyst

In order to acquire the crystal structure of samples, X-ray powder diffraction (XRD) was obtained by Bruker D8 employing Cu-Kα radiation (1.54056 Å) with an operating voltage of 40 kV and a beam current of 40 mA. The morphologies of samples were obtained on a Hitachi S-4800 scanning electron microscope with an accelerating voltage of 15 kV, and high-resolution transmission electron microscopes (HRTEM) with an accelerating voltage of 200 kV (JEOL JEM-2010F electron microscope). The element component of catalysts was measured by the energy disperse X-ray spectrum (EDS, EDAXTLS). The surface components and valence states of samples were characterized by X-ray photoelectron spectra (XPS) (Thermo Fisher Scientific ESCALAB250Xi) employing Al Kα (1486.6 eV).

H_2 temperature-programmed reduction (H_2-TPR) was implemented on Thermo TPDRO 1100 series to investigate the reducibility of catalysts. Typically, 50 mg of samples were pretreated firstly in the N_2 stream at 120°C for 60 min in a quartz reactor with a flow rate of 30 mL/min, and then they were cooled to room temperature. The gas feed was switched to 5% of the H_2-N_2 gas mixture. The amount of H_2 consumption was monitored by a thermal conductivity detector (TCD). The TPR profile was recorded with temperature programming at a rate of 10°C min^{-1} from 50 to 500°C.

For O_2 temperature-programmed desorption (O_2-TPD), about 100 mg of catalyst was pretreated in a He stream at 120°C for 60 min with a flow rate of 30 mL/min. The temperature was reduced to room temperature and subsequently saturated with 10% O_2 (balanced with He) for 60 min, then purged by a pure He stream for 60 min. Finally, the temperature was heated to 600°C at a rate of 10 °C/min.

2. The test of catalytic performance

The catalytic performance of catalysts was evaluated by CO oxidation. In a typically process, 100 mg of catalyst without any pretreatment was loaded in a fixed-bed quartz reactor quartz reactor (30 cm in length, 8 mm in i.d.) at atmospheric pressure. The mixed feed gas contained with 1 vol% CO and 20 vol% O_2 in N_2 balanced with a flow rate of 50 ml min^{-1} (space velocity = 30000 ml•$gcat^{-1}$•h^{-1}) was applied to the reactor. The variety of gas concentration was monitored with an online gas chromatography

system (Agilent 7890B) equipped with a TCD. The CO conversion rate was calculated based on the following formula.

$$\text{CO conversion (\%)} = \frac{[\text{CO}]_{\text{in}} - [\text{CO}]_{\text{out}}}{[\text{CO}]_{\text{in}}} \times 100\%$$

3. DFT Calculations

Spin-polarized density functional theory (DFT) calculations were performed using the Quantum ESPRESSO, coupled with the generalized gradient approximation (GGA) of the Perdew–Burke–Ernzerhof (PBE) for the exchange correlation functional. The plane-wave cutoff energy is 400 eV. The (111) plane of CuO and (311) plane of $Cu_{1.5}Mn_{1.5}O_4$ were chosen for the theoretical calculations according to the HRTEM and XRD results. The 3D periodic slabs of CuO, $Cu_{1.5}Mn_{1.5}O_4$, and $CuO_x/Cu_{1.5}Mn_{1.5}O_4$ surfaces were chosen to interact with CO and O_2 molecules. The three slab models including four atomic layers were constructed with a vacuum space of 20 Å and 4 × 4 × 1 Monkhorst–Pack grid for surface calculations. During geometry optimization, the atoms in the bottom two atomic layers were fixed at their bulk-truncated positions and the top two atomic layers along with the adsorbates were fully relaxed. The adsorption configurations of CO and O_2 molecules on model atomic configurations were explored. The adsorption energy (E_{ads}) was defined by E_{ads} = - ($E_{CO\text{-}slab}$ - E_{slab} - E_{CO}), where, $E_{CO\text{-}slab}$, $E_{slab,}$ and E_{CO} are total energy for the CO-slab complex, the isolated slab, and the isolated CO molecule, respectively.

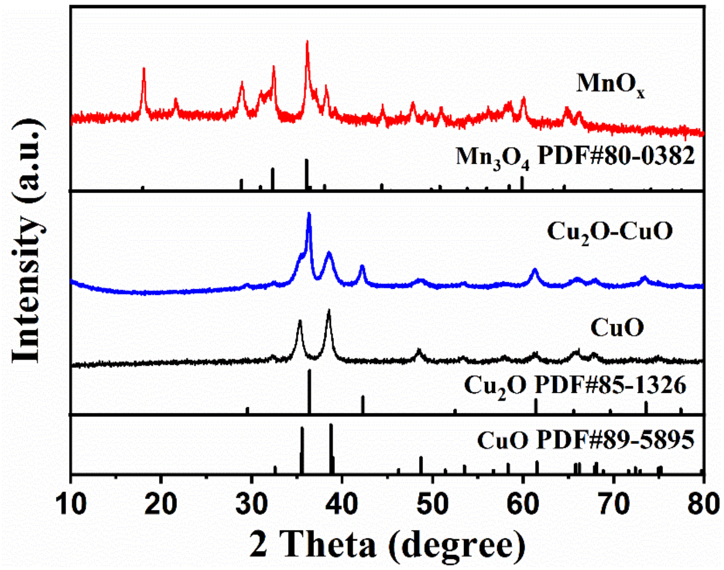

Figure S1. XRD patterns of as-synthesized MnO_x, Cu_2O-CuO, and CuO.

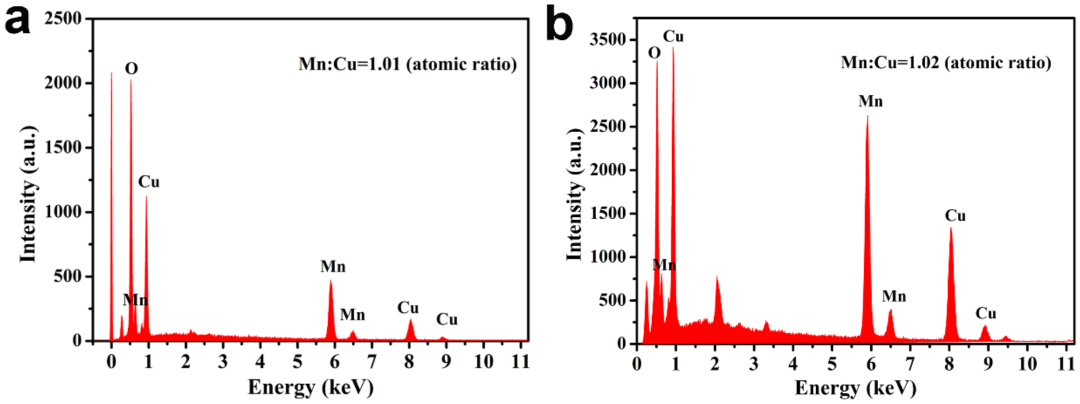

Figure S2. EDS spectra of the CuO_x-MnO_x nanocomposites: (a) $Cu_{1.5}Mn_{1.5}O_4$ and (b) $CuO_x/Cu_{1.5}Mn_{1.5}O_4$.

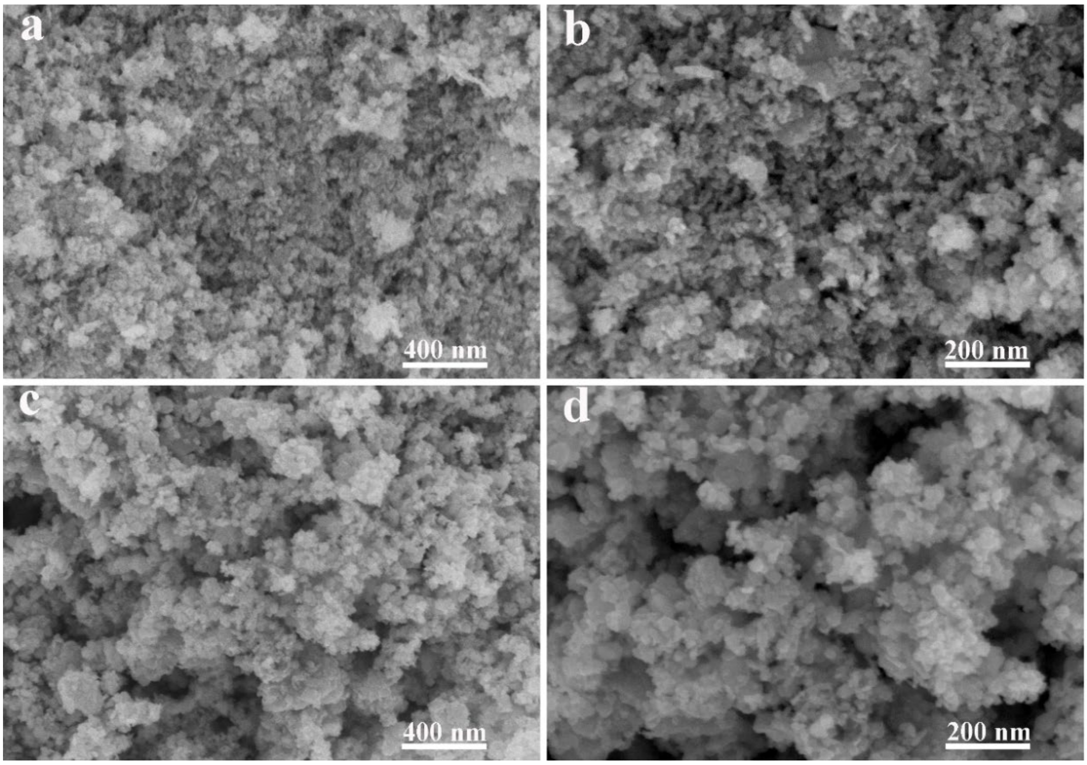

Figure S3. FESEM images for CuO_x-MnO_x nanoparticles with different Cu/Mn mole ratio: (a-b) Cu/Mn = 1:2 and (c-d) Cu/Mn = 2:1.

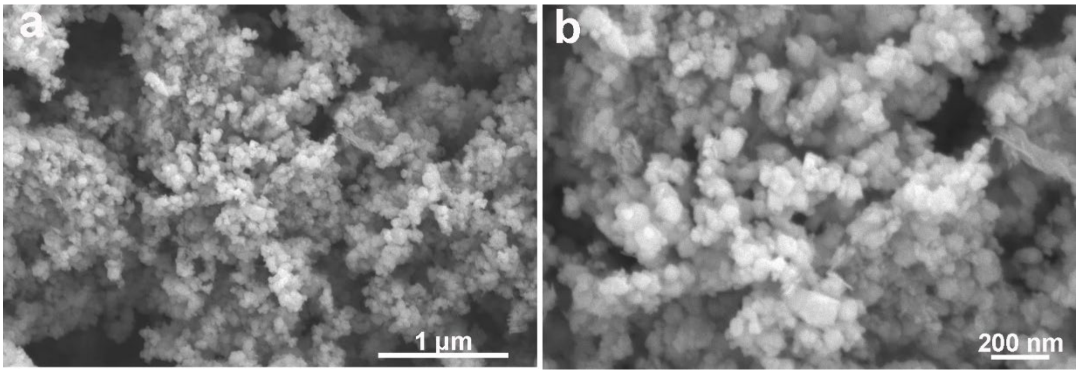

Figure S4. FESEM images for MnO_x nanoparticles.

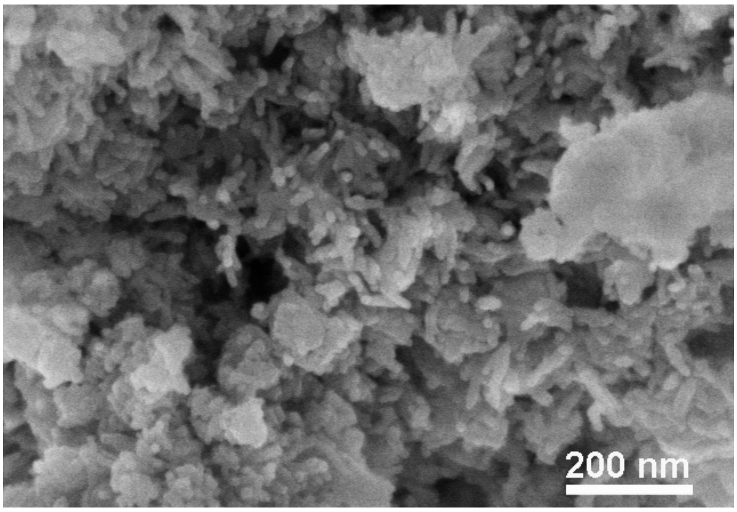

Figure S5. FESEM image for Cu_2O-CuO nanocomposite.

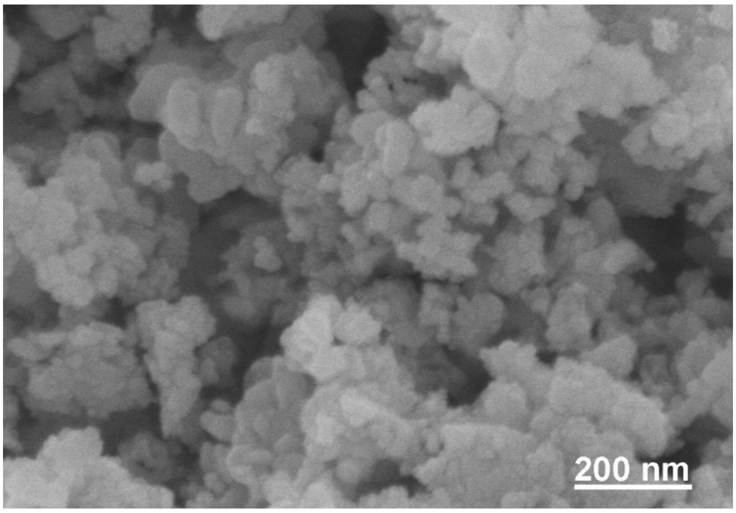

Figure S6. FESEM image for CuO nanoparticles.

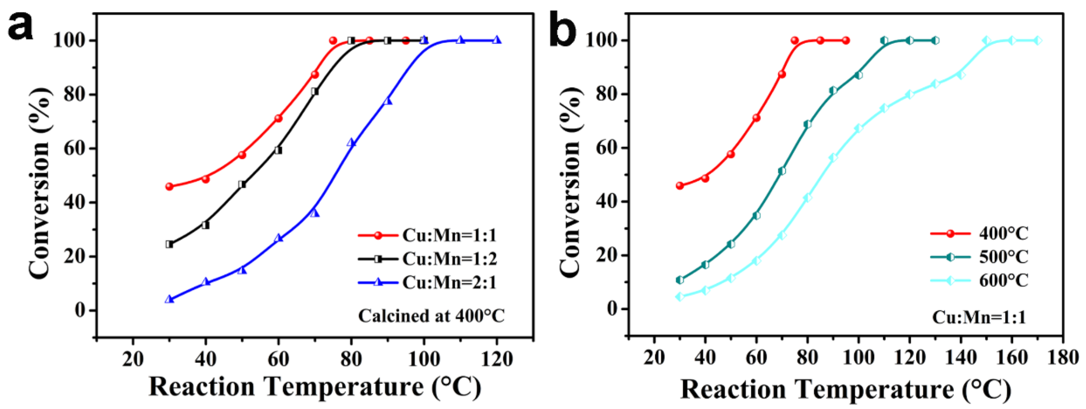

Figure S7. The CO conversion performances over a series of CuO_x-MnO_x nanocomposites with different (a) Cu/Mn mole ratio and (b) calcination temperature.

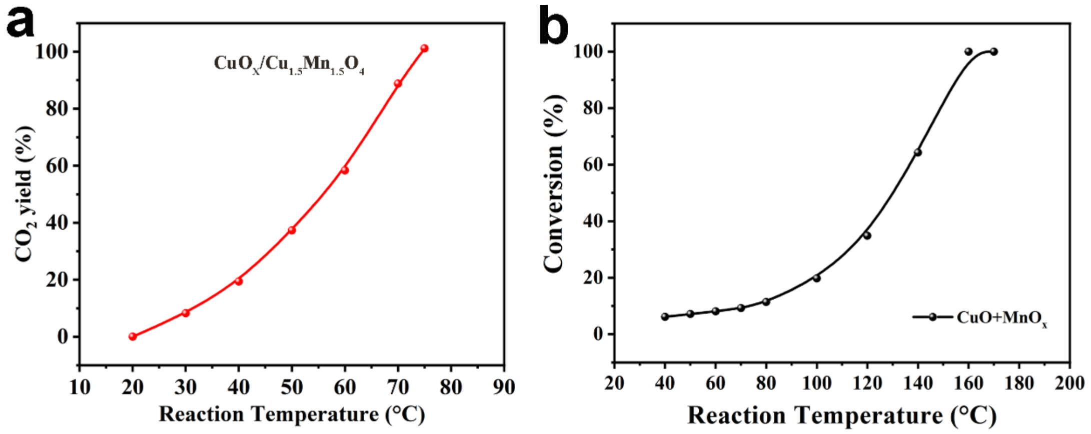

Figure S8. (a) CO_2 yield in the CO oxidation of $CuO_x/Cu_{1.5}Mn_{1.5}O_4$; (b) the CO conversion performances of physical mixing of CuO_x+MnO_x catalyst for CO oxidation.

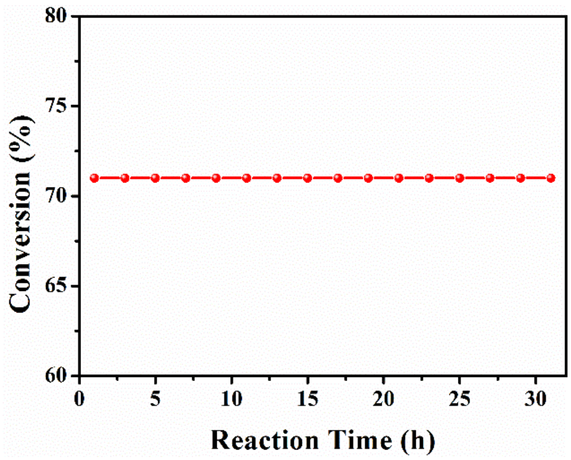

Figure S9. The stability test of $CuO_x/Cu_{1.5}Mn_{1.5}O_4$ sample for CO oxidation.

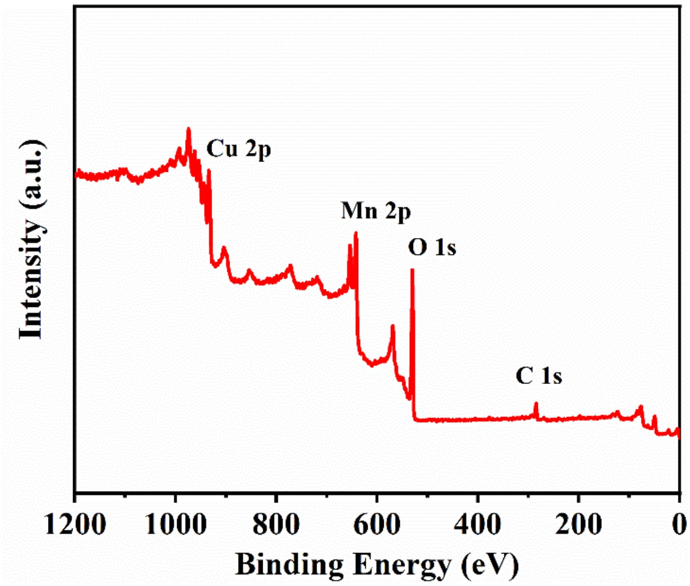

Figure S10. The survey of XPS spectrum of $CuO_x/Cu_{1.5}Mn_{1.5}O_4$.

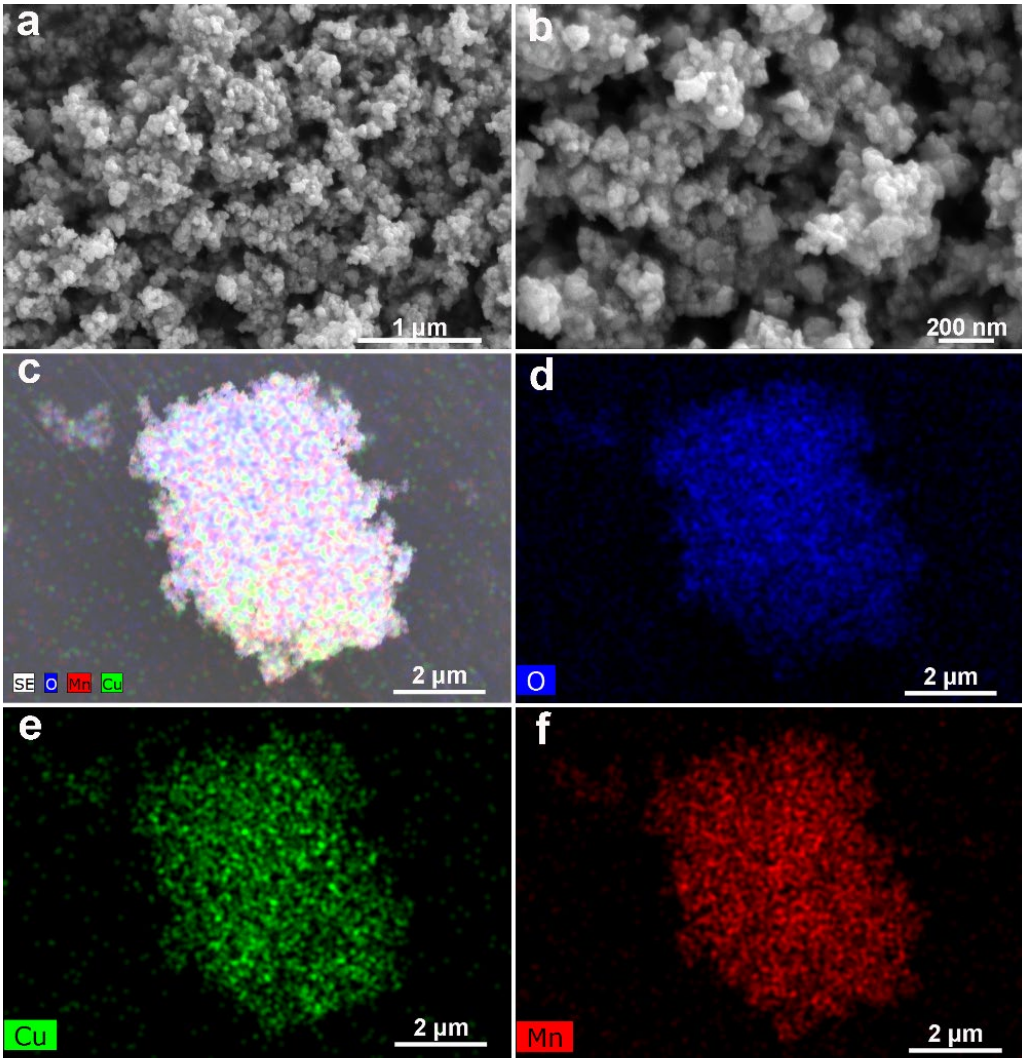

Figure S11. (a-b) FESEM images, and (c-f) the corresponding element mapping patterns of $Cu_{1.5}Mn_{1.5}O_4$ sample.

Table S1. Catalytic activity of CO oxidation with CuO_x-MnO_x nanocomposites obtained from different preparation method reported in previous literature.

Sample	Method	Amount of catalyst (mg)	Feed gas composition	Flow rate ($mL \cdot min^{-1}$)	T_{50} (°C)	T_{100} (°C)	Ref.
CuO/Mn_2O_3	Sol–gel method	75	$CO/O_2/Ar$=8/20/72	25	81	98	[1]
Cu–Mn/CeO_2	Wet co-impregnation	9300	CO/Ar=0.4/99.6	300	95.7	145.6	[2]
Mn_3O_4/CeO_2	Hydrothermal method	100	$CO/O_2/He$ = 1/4/95	-	103	194 (T_{90})	[3]
Cu-Mn-O catalysts	Spray pyrolysis	100	CO/O_2/ He =1/20/79	10	32	63	[4]
Mn-Cu-Co oxide	Chemical Solution method	100	$CO/O_2/Ar$=1/20/79	30	34	75 (T_{90})	[5]
CuO/MnO_2	Solution method	50	CO/Air=1/99	20	-	110	[6]
$CuO_x/Cu_{1.5}Mn_{1.5}O_4$ nanocomposite	Solid-state chemical method	100	$CO/O_2/N_2$ = 1/20/79	50	41	75	This work

Table S2. XPS results of different samples.

Sample	Cu^{+} (%)[a]	Cu^{2+} (%)[b]	Mn^{3+} (%)[c]	Mn^{4+} (%)[d]	O_{α} (%)[e]	O_{β} (%)[e]	O_{γ} (%)[e]
$Cu_{1.5}Mn_{1.5}O_4$	39.43	60.57	77.00	23.00	76.96	17.42	5.62
$CuO_x/Cu_{1.5}Mn_{1.5}O_4$	24.81	75.19	74.79	24.21	74.89	19.41	5.69

[a] Cu^{+} proportion were estimated by (Cu^{+}/Cu^{2+} + Cu^{+}) × 100% from XPS;

[b] Cu^{2+} proportion were estimated by (Cu^{2+}/Cu^{2+} + Cu^{+}) × 100% from XPS;

[c] Mn^{3+} proportion were estimated by (Mn^{3+}/Mn^{4+} + Mn^{3+}) × 100% from XPS;

[d] Mn^{4+} proportion were estimated by (Mn^{4+}/Mn^{4+} + Mn^{3+}) × 100% from XPS;

[e] O_{α}, lattice oxygen; O_{β}, surface adsorbed oxygen; O_{γ}, chemisorbed water and/or carbonates.

Table S3. The peak areas of different samples integrated from corresponding H_2-TPR profiles.

Sample	Peak α	Peak β	Peak γ
$Cu_{1.5}Mn_{1.5}O_4$	0.54	4.43	17.61
$CuO_x/Cu_{1.5}Mn_{1.5}O_4$	1.24	3.39	17.97

References

1. Rastegarpanah, A.; Rezaei, M.; Meshkani, F., Dai, H. 3D ordered honeycomb-shaped $CuO \cdot Mn_2O_3$: Highly active catalysts for CO oxidation. *Mol. Catal.* **2020**, *485*, 1108201-1108206.
2. Lin, J.; Guo, Y.; Chen, X.; Li, C.; Lu, S., Liew, K.M. CO Oxidation over Nanostructured Ceria Supported Bimetallic Cu-Mn Oxides Catalysts: Effect of Cu/Mn Ratio and Calcination Temperature. *Catal. Lett.* **2018**, *148* (1), 181-193.
3. Jampaiah, D.; Velisoju, V.K.; Devaiah, D.; Singh, M.; Mayes, E.L.H.; Coyle, V.E.; Reddy, B.M.; Bansal, V., Bhargava, S.K. Flower-like Mn_3O_4/CeO_2 microspheres as an efficient catalyst for diesel soot and CO oxidation: Synergistic effects for enhanced catalytic performance. *Appl. Surf. Sci.* **2019**, *473*, 209-221.
4. Zhou, Y.; Liu, X.; Wang, K.; Li, J.; Zhang, X.; Jin, X.; Tang, X.; Zhu, X.; Zhang, R.; Jiang, X., Liu, B. Porous Cu-Mn-O catalysts fabricated by spray pyrolysis method for efficient CO oxidation. *Results in Physics* **2019**, *12*, 1893-1900.
5. Choi, K.-H.; Lee, D.-H.; Kim, H.-S.; Yoon, Y.-C.; Park, C.-S., Kim, Y.H. Reaction Characteristics of Precious-Metal-Free Ternary Mn–Cu–M (M = Ce, Co, Cr, and Fe) Oxide Catalysts for Low-Temperature CO Oxidation. *Ind. Eng. Chem. Res.* **2016**, *55* (16), 4443-4450.
6. Qian, K.; Qian, Z.X.; Hua, Q.; Jiang, Z.Q., Huang, W.X. Structure–activity relationship of CuO/MnO_2 catalysts in CO oxidation. *Appl. Surf. Sci.* **2013**, *273*, 357-363.
